# Supplementary material for: Risky choice: Probability weighting explains independence axiom violations in monkeys
Source: J Risk Uncertain. 2022 Jul 22;65(3):319–51. doi: 10.1007/s11166-022-09388-7 (PMC9840594; doi:10.1007/s11166-022-09388-7)
Supplement: Supplementary file 1 — Supplementary file1 (DOCX 1949 KB) [file 11166_2022_9388_MOESM1_ESM.docx]

**Risky choice: probability weighting explains Independence Axiom violations in monkeys**

**Simone Ferrari-Toniolo*, Leo Chi U Seak*, Wolfram Schultz**

Department of Physiology, Development and Neuroscience

University of Cambridge, Cambridge, UK

Corresponding author email: Simone.ferraritoniolo@gmail.com


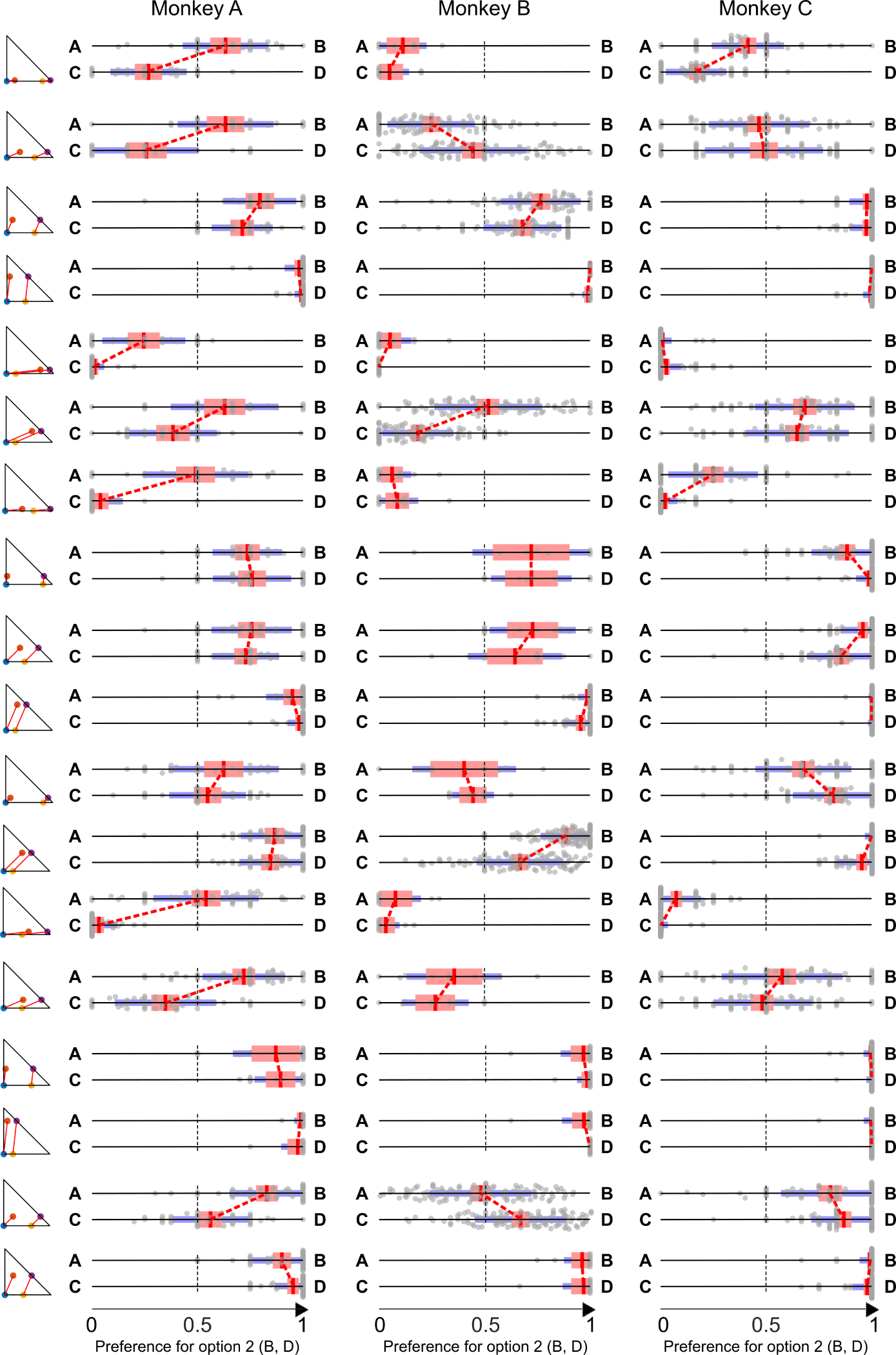


**Supplementary Fig. 1.** *Preference Changes* S for all common consequence tests. For conventions, see Fig. 2.


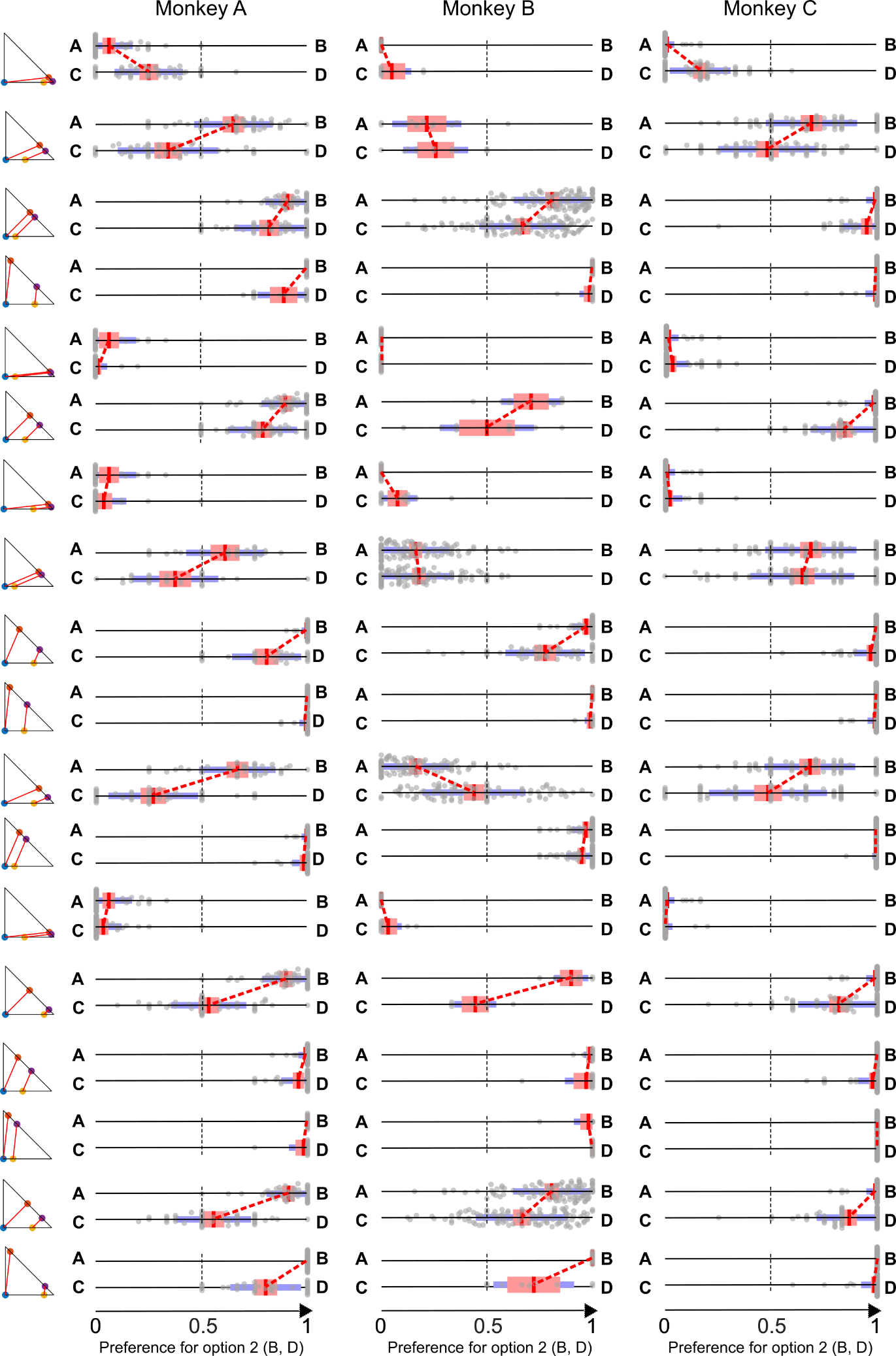


**Supplementary Fig. 2.** *Preference Changes* S for all common ratio tests. For conventions, see Fig. 2.


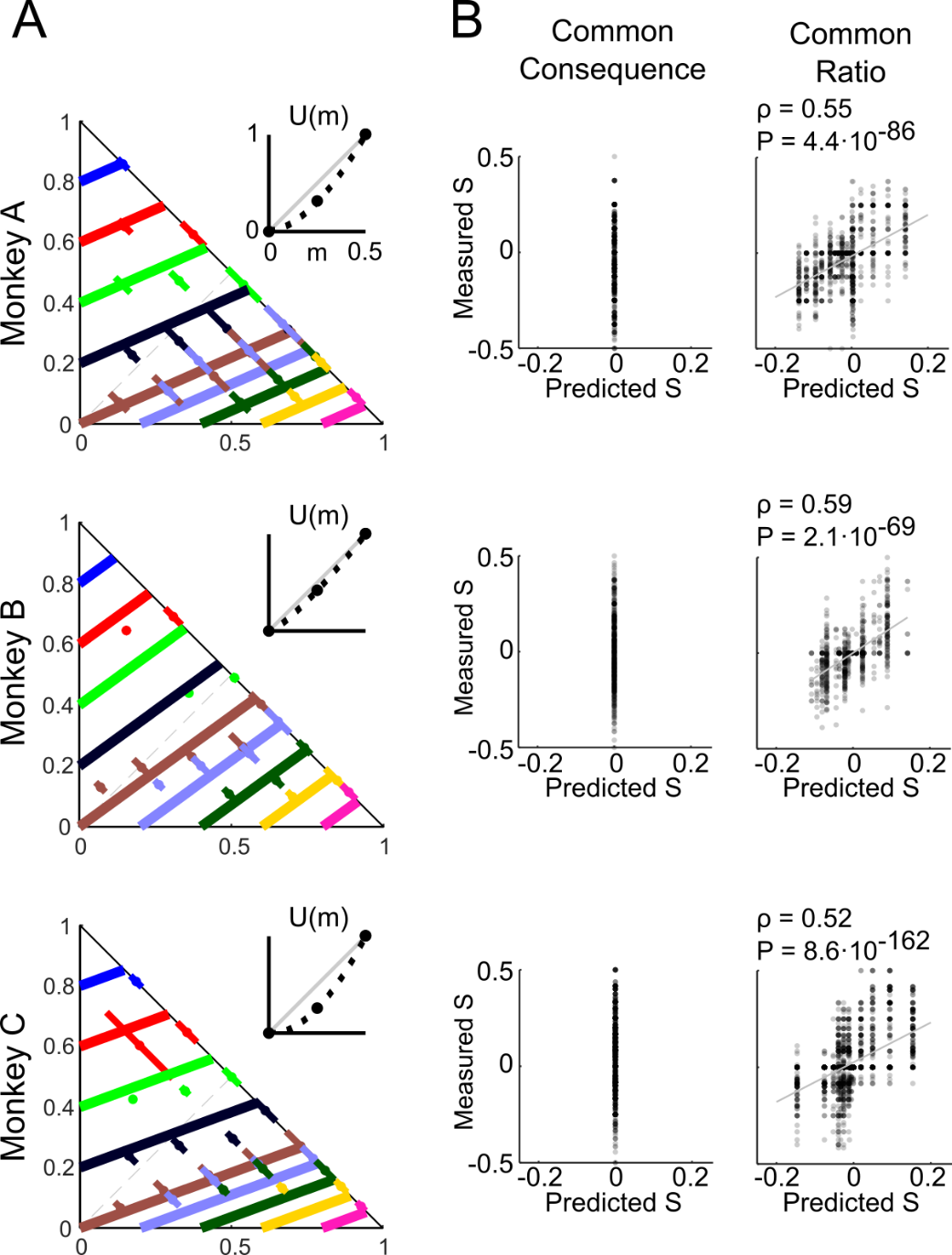


**Supplementary Fig. 3.** Expected Utility Theory (EUT) modeling failed to explain the common consequence (CC) test but can predict the Preference Changes of the common ratio (CR) test to some degree. For conventions, see Fig. 6.


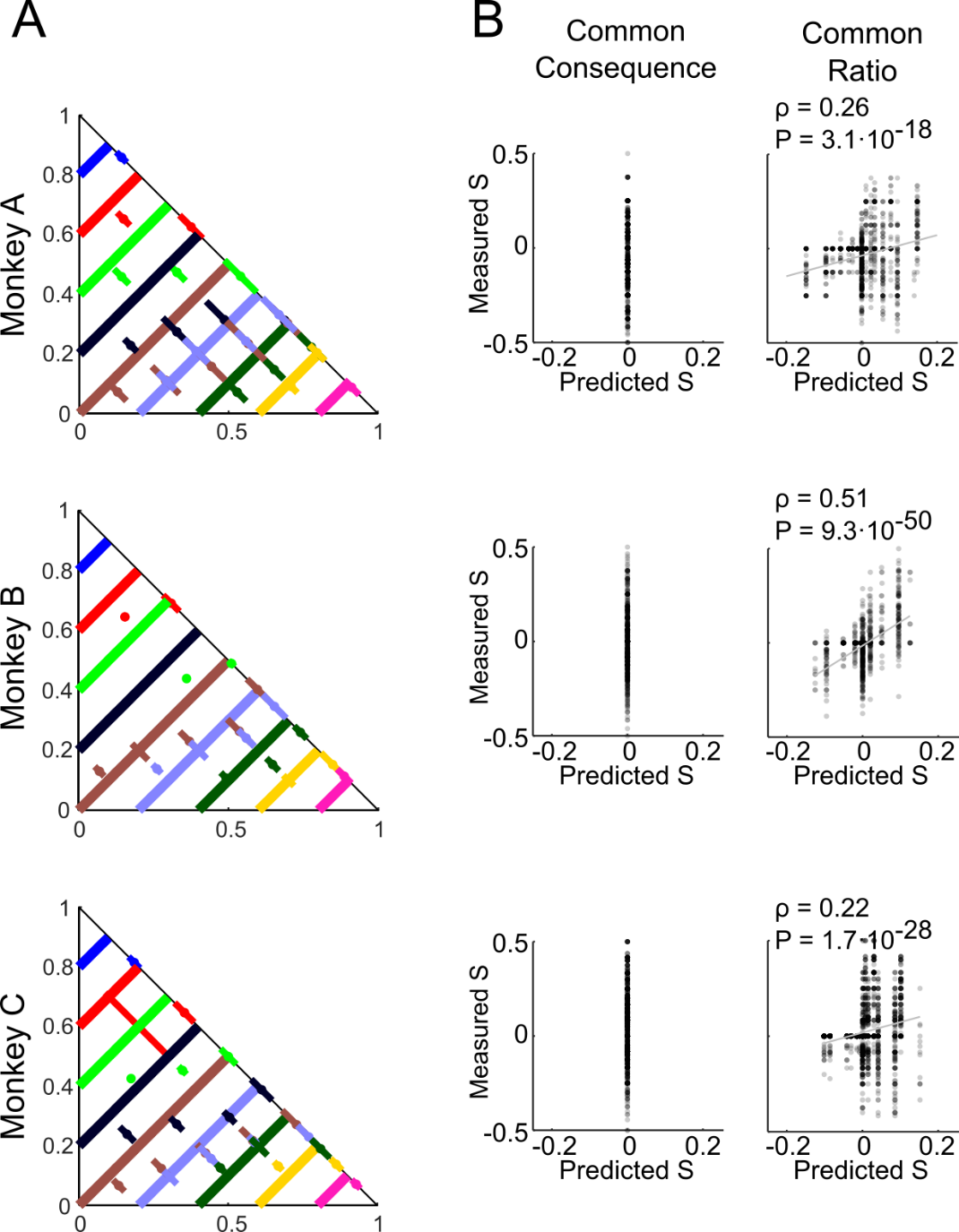


**Supplementary Fig. 4.** Expected Value (EV) modeling failed to explain the common consequence (CC) test but can predict the Preference Changes of the common ratio (CR) to some degree. For conventions, see Fig. 6.


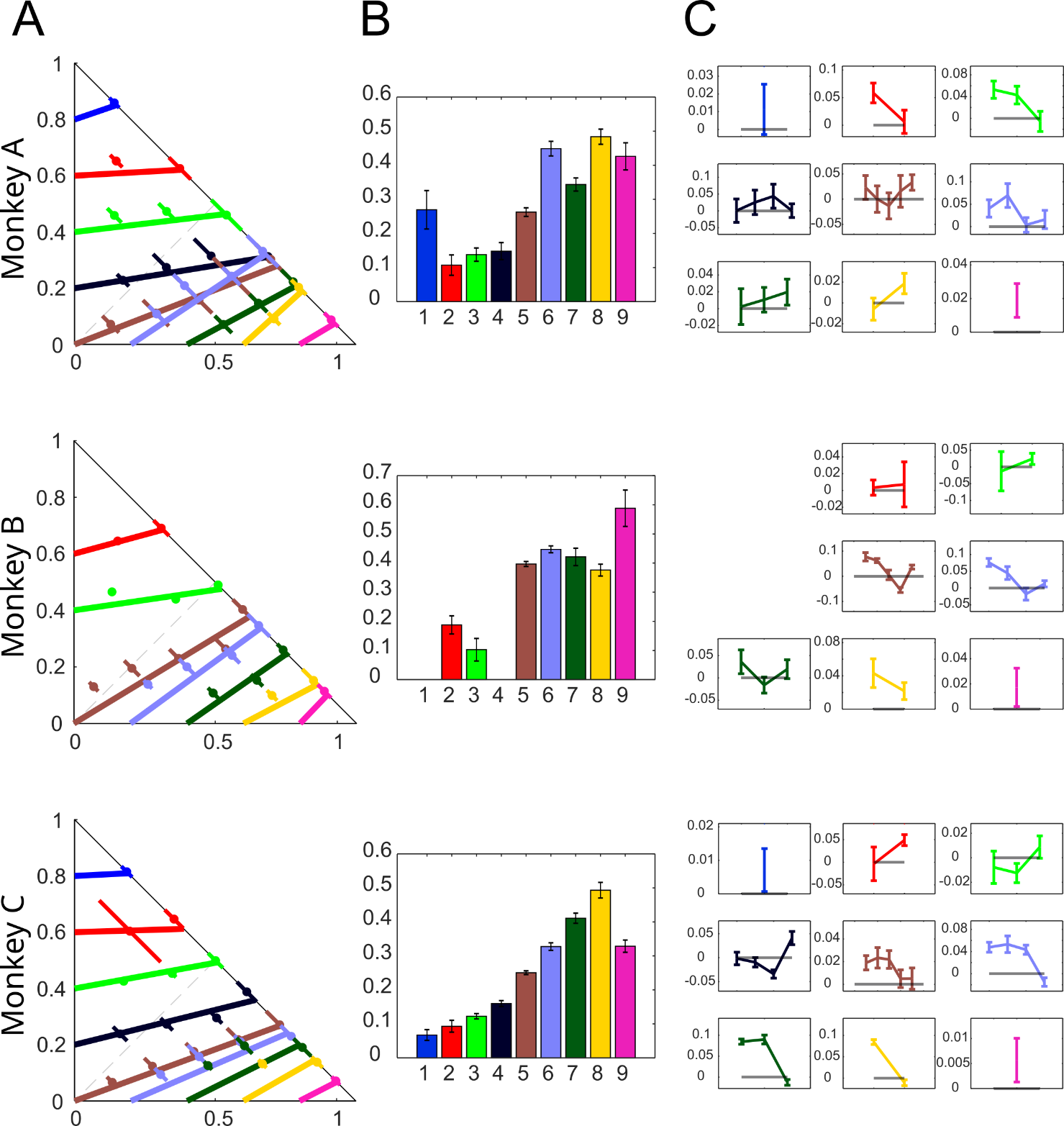


**Supplementary Fig. 5**. Out-of-sample test for linearity and parallelism of indifference curves in the Marschak-Machina triangle. (A) Indifference curves estimated with linear least-squares and out-of-sample indifference points. Dots represent the mean of indifference points across all sessions (that fall within the same indifference curve with the same color); lines show Standard Deviation (SD) of IPs across all sessions. (B) Bar graphs showing significant differences in slope of the indifference curves estimated with linear least-squares (mean ± SEM; one-way ANOVA p < 0.001 for all three animals). The nine colors correspond to the nine indifference curves being tested. (C) Line plots showing significant residuals between indifference points and estimated indifference curves (mean ± SEM; p<0.05; one-sample t-test against indifference curves, represented by grey lines). The y-axis represents the residuals (0 = same as estimated indifference curves), and the x-axis represents the position of the indifference points in the Marschak-Machina triangle.
